# Development and validation of next-generation sequencing panel for personalized *Helicobacter pylori* eradication treatment targeting multiple species

**DOI:** 10.3389/fcimb.2024.1379790

**Published:** 2024-08-29

**Authors:** Byung-Joo Min, Myung-Eui Seo, Jung Ho Bae, Ji Won Kim, Ju Han Kim

**Affiliations:** ^1^ Forensic DNA Division, National Forensic Service Seoul Institute, Seoul, Republic of Korea; ^2^ Seoul National University Biomedical Informatics (SNUBI), Department of Biomedical Sciences, Seoul National University College of Medicine, Seoul, Republic of Korea; ^3^ Department of Internal Medicine and Healthcare Research Institute, Healthcare System Gangnam Center, Seoul National University Hospital, Seoul, Republic of Korea; ^4^ Department of Internal Medicine, Seoul Metropolitan Government - Seoul National University Boramae Medical Center, Seoul, Republic of Korea; ^5^ Department of Internal Medicine, Seoul National University College of Medicine, Seoul, Republic of Korea; ^6^ Seoul National University Biomedical Informatics (SNUBI), Division of Biomedical Informatics, Seoul National University College of Medicine, Seoul, Republic of Korea

**Keywords:** personalized *Helicobacter pylori* eradication treatment, individual antibiotic resistance profile, antibiotic resistance, proton-pump inhibitor metabolic phenotype, multispecies integrated next-generation sequencing panel

## Abstract

**Introduction:**

The decreasing *Helicobacter pylori* eradication rate is primarily attributed to antibiotic resistance, and further exacerbated by uniform drug administration disregarding a host’s metabolic capability. Consequently, applying personalized treatment based on antibiotic resistance-associated variants and the host’s metabolic phenotype can potentially increase the eradication rate.

**Method:**

A custom next-generation sequencing panel for personalized *H. pylori* eradication treatment (NGS-PHET) was designed which targeted the regions for amoxicillin, clarithromycin, metronidazole, tetracycline, and levofloxacin-resistance in *H. pylori* and human proton-pump inhibitor (PPI) metabolism. The libraries were constructed following customized methods and sequenced simultaneously. The customized framework criteria, grounded in previously reported antibiotic resistance associated variants and the host’s PPI metabolism, was applied to the NGS-PHET results and suggested a personalized treatment for each subject, which was validated through each subject’s actual eradication outcome.

**Results:**

Both previously reported and novel variants were identified from *H. pylori* sequencing results. Concurrently, five *CYP2C19* homozygous extensive metabolizers and three *CYP3A4* intermediate metabolizers were identified. Among the total of 12 subjects, clarithromycin triple therapy was suggested for five subjects, bismuth quadruple therapy was suggested for six subjects, and rifabutin triple therapy was suggested for one subject by following the customized framework criteria. The treatment suggestion for nine of the 12 subjects was consistent with the treatment that each subject achieved eradication with.

**Discussion:**

Applying the methodology using the NGS-PHET and customized framework helps to perform eradication treatment quickly and effectively in most patients with antibiotic-resistant *H. pylori* strains, and is also useful in research to find novel antibiotic-resistance candidates.

## 1 Introduction

*Helicobacter pylori* has been widely acknowledged as a highly significant human pathogen due to its capacity to induce severe diseases and infect approximately 50% of the global population (Matsukura et al., 1995; McColl, 2010). As *H. pylori* infection is a worldwide human-to-human infectious disease, the need for eradication treatment has emerged for individuals requiring treatment or prevention of diverse gastrointestinal diseases associated with *H. pylori* (Jung et al., 2021). Triple therapy that consists of a proton pump inhibitor (PPI) in combination with two antibiotics has been used as the first-line treatment. However, as the cure rate of traditional triple therapy is 80% or lower (Lee et al., 2022), alternative methods such as treatment with new antibiotics or personalized therapy are required (Lee et al., 2014).

A predominant factor for treatment failure is the resistance to major antibiotics due to *H. pylori* mutations (Hu et al., 2016; Savoldi et al., 2018; Pohl et al., 2019). There are *H. pylori* strains with various trends of resistance to each major antibiotic in each country or region, and the resistance trend changes are observed over time even within one region (Lee et al., 2013; Shin et al., 2016; Lee et al., 2019; Malfertheiner et al., 2022). Therefore, it is evident that prescriptions altering the type or dosage of antibiotics without considering the genetic polymorphism of the *H. pylori* strain yield only short-term efficacy. Another consideration in treatment failure is the polymorphism of PPI metabolism-associated human genes (Malfertheiner et al., 2022). The most commonly used PPIs such as omeprazole and lansoprazole are mainly metabolized by the cytochrome P450 family 2 subfamily C member 19 [*CYP2C19* [MIM: 124020] (OMIM, 2019b)] and cytochrome P450 family 3 subfamily A member 4 [*CYP3A4*, [MIM: 124010] (OMIM, 2019a)] (Gonzalez et al., 2003). According to the Maastricht VI/Florence consensus report, *CYP2C19* polymorphism-associated metabolic ability significantly influences response to PPI and extended metabolizers (EMs) are recommended to increase the success rate of eradication by administering higher PPI doses to adequately control gastric pH (Malfertheiner et al., 2022). In addition, by categorizing patients by *CYP2C19* polymorphism and administering omeprazole, a previous study noted a higher eradication rate in poor metabolizers (PMs) than EMs (Lin et al., 2017). These reports suggest that both *H. pylori* variants and host variants must be considered to eliminate the factors of failure and increase the eradication success rate effectively.

Two representative methods currently used for testing *H. pylori* are culture-based testing and culture-free molecular testing (Hulten et al., 2021). Successful performance of culture-based testing requires appropriate treatment of biopsy, complex sample transport conditions, and an experienced laboratory (Hulten et al., 2021). Furthermore, the method is rarely employed in clinical practice because of the difficulty of culture and the extended duration required for the results. In comparison, molecular-based testing, such as a polymerase chain reaction (PCR) based assay, has the advantage of being able to provide multiple antibiotic resistance-associated mutations within a week. Nevertheless, most molecular tests currently in use have limitations in that they are designed to detect only a single or small set of mutations in a particular gene or limited area (Hulten et al., 2021). There is an impressive amount of genetic diversity among *H. pylori* strains driven by high mutation rates, frequent recombinant events, random genetic drift as well as positive Darwinian selection and fixation of base substitution, requiring improvement on the limited target range and accuracy of modern molecular tests (Torres-Morquecho et al., 2010). Moreover, since the host genetic polymorphism is not considered during each culture-based testing and when the culture-free PCR assay for *H. pylori* is applied, additional cost and time are needed to analyze the host genome. Molecular tests that apply next-generation sequencing (NGS) techniques have been suggested as an alternative to address the limitations of these methods (Pohl et al., 2019). When NGS-based molecular testing is applied to clinical practice, it is possible to concurrently test multiple large target areas. Additionally, there is an evaluation that other methods may be preferable over *H. pylori* culture-based diagnostics, as related technologies have been improved to accurately identify mutations even in small amounts of DNA (Mommersteeg et al., 2023; Ali and AlHussaini, 2024). Consequently, studies applying NGS technology to decode *H. pylori* genetic information are continuously reported. Furthermore, to propose a personalized eradication treatment using various information provided by the patient, an eradication treatment guideline based on the information is required. Currently, eradication treatment guidelines founded on the prior eradication treatment outcomes are described, but guidelines grounded in the results of molecular testing have not been established except for clarithromycin (Malfertheiner et al., 2022). Therefore, to suggest a personalized eradication treatment using a large amount of data derived from an NGS-based molecular test, a customized eradication treatment framework based on that data is needed.

**Figure 1:**
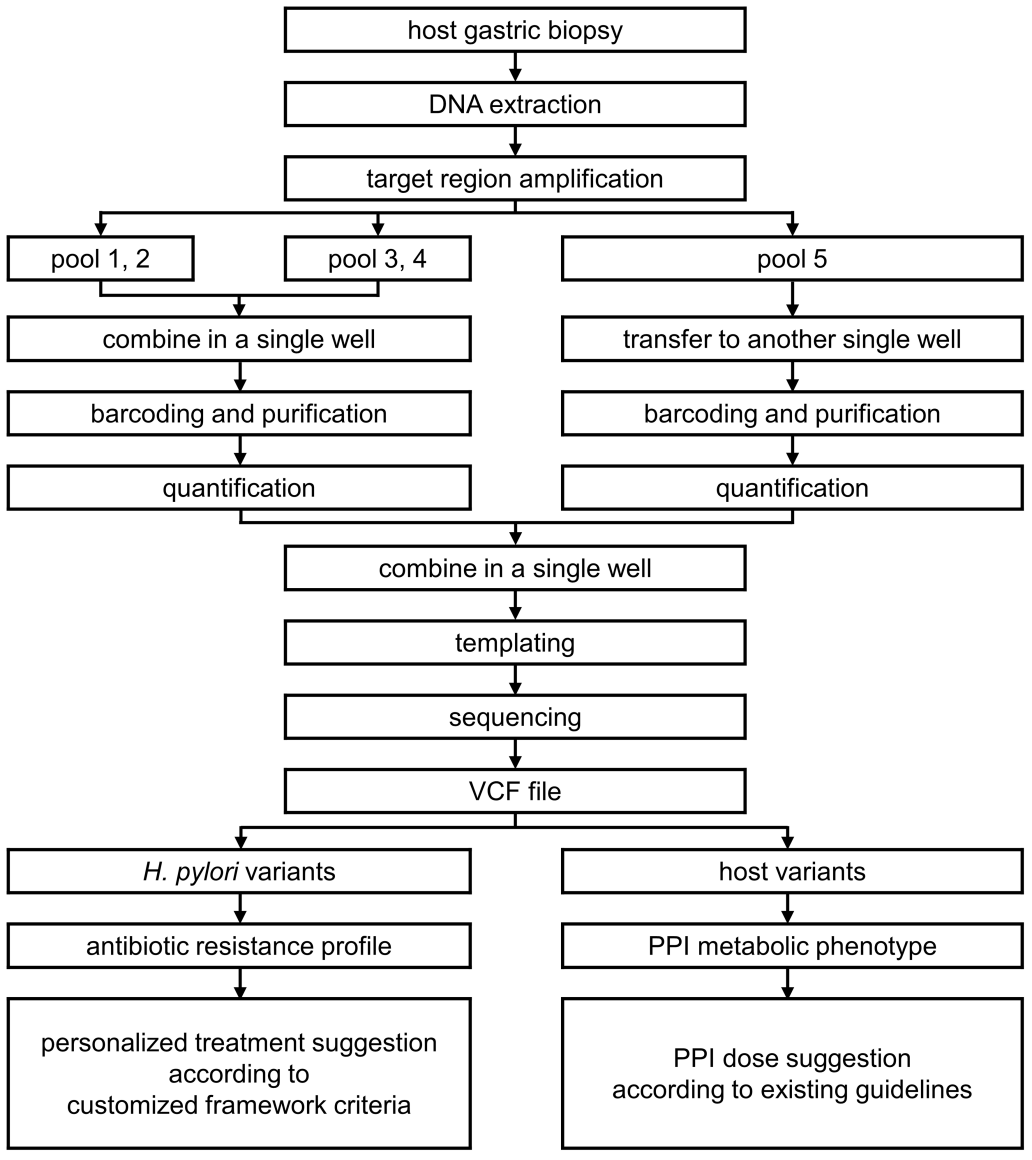
Methodologies used for custom NGS panel for personalized *H. pylori* eradication treatment (NGS-PHET).

In this study, we developed a custom NGS panel for personalized *H. pylori* eradication treatment (NGS-PHET), which is a multi-species integrated NGS panel that targets antibiotic resistance-related *H. pylori* genes and PPI metabolism-associated host genes and designed customized framework criteria on the basis of previously reported genetic variants associated with antibiotic resistance associated with *H. pylori* and the PPI metabolizing ability of the host. The methodology was applied to DNA extracted from a patient’s gastric biopsy to analyze *H. pylori* and host genes simultaneously, and individual *H. pylori* eradication treatments were proposed (**Figure 1**). In addition, each proposal was validated by comparing it with the patient’s actual antibiotic treatment outcome.

## 2 Materials and methods

### 2.1 Gastric biopsy sample for NGS

Gastric biopsies were obtained from subjects who voluntarily completed a consent form to participate in the study and were followed up until eradication treatment results were confirmed. These were sourced from patients who became candidates for *H. pylori* eradication treatment in routine clinical settings in Korea and were subsequently treated with the standard *H. pylori* eradication treatment in Korea at Seoul Metropolitan Government - Seoul National University Boramae Medical Center (SMG-SNU Boramae Medical Center) between October 2018 and September 2020 (Kim et al., 2014). *H. pylori* eradication treatment candidates were selected using following inclusion criteria: (1) patients aged 19 years or older who underwent gastroscopy due to gastrointestinal symptoms (abdominal pain, nausea, indigestion, heartburn, etc.), (2) patients who showed peptic ulcers, chronic atrophic gastritis, and intestinal gastritis at the gastroscopy, (3) patients who tested positive for *H. pylori* through gastric biopsy obtained during the gastroscopy by rapid urease test (ASAN Helicobacter Test kit, Asan Pharm. CO., LTD., Gyeonggi-do, Republic of Korea). The following patients were excluded: (1) individuals with a history of prior treatment for *H. pylori* eradication, and (2) individuals with a history of prior treatment for malignant tumors. Positive controls for the NGS panel test were secured by applying the same criteria as the inclusion criteria while the negative control was a biopsy sample identified as *H. pylori* negative by a rapid urease test. For this study, we enrolled biopsies from three positive controls, one negative control, and 12 subjects.

For *H. pylori* eradication treatment, standard triple therapy consisting of PPI (lansoprazole), amoxicillin, and clarithromycin (PAC) was administered for 7 days as the first-line treatment. Four weeks after completing the treatment, a rapid urease test using gastric biopsy and a ^13^C urea breath test (UBT) were conducted for the confirmation of *H. pylori* eradication. Confirmation of *H. pylori* eradication was established when both tests revealed negative results. For patients who exhibited first-line treatment failure, bismuth-containing quadruple therapy using PPI (lansoprazole), bismuth, metronidazole, and tetracycline (PBMT) was implemented for a duration of 7 days as the second-line treatment. In cases where patients did not achieve eradication with the second-line treatment, a third-line treatment including PPI (lansoprazole), amoxicillin, and quinolone (levofloxacin) (PAL) was administered for 7 days. The rapid urease test and the ^13^C UBT were performed 4 weeks after completing each treatment to identify the status of *H. pylori* eradication.

### 2.2 Selecting target regions associated with antibiotic resistance of *H. pylori* for panel design

*H. pylori* genes associated with resistance to the antibiotics that are used for the first-line, second-line, and third-line treatments were selected for the NGS panel. At the SMG-SNU Boramae Medical Center, amoxicillin, clarithromycin, lansoprazole, levofloxacin, metronidazole, and tetracycline were included in the eradication therapies.

As multiple variants in penicillin-binding modules of penicillin-binding protein 1 (*pbp1*) are responsible for amoxicillin resistance (Okamoto et al., 2002; Nishizawa and Suzuki, 2014), a whole sequence of *pbp1* was included in the target region. For clarithromycin, A2142G or C, A2143G, and A2144G of *HPrrna23S* were included because the variants have been consistently confirmed as the reason for resistance by multiple studies (Nishizawa and Suzuki, 2014). The variants in the quinolones resistance-determining region (QRDR) of *gyrA* and *gyrB* confer levofloxacin resistance (Moore et al., 1995; Nishizawa et al., 2009; Nishizawa and Suzuki, 2014). The entire sequence of *gyrA* and *gyrB* were included in the panel design. As inactivation of oxygen-insensitive NADPH nitroreductase (RdxA), NADPH-flavin-oxidoreductase (FrxA), and/or ferredoxin-like enzymes (FrxB) are associated with metronidazole resistance (Kwon et al., 2001; Lee et al., 2018), the complete genome sequence of *rdxA*, *frxA*, and *frxB* were comprised in the panel. The triple point-mutations at positions 965–967 of *HPrrna16S* are predominantly responsible for tetracycline resistance (Pioletti et al., 2001; Bauer et al., 2004; Chukwudi, 2016), and the panel was designed to target the region. Cytotoxin-associated gene A (*cagA*) is the most important virulence factor of *H. pylori* and the only protein that is translocated into host cells by adherent *H. pylori* (Yamazaki et al., 2005; Vital et al., 2022). Accordingly, the amplicons targeting *cagA* were included in the panel.

In consequence, the following genes were selected for the custom NGS panel: *HPrrna23S*, *pbp1*, *HPrrna16S*, *rdxA*, *frxA*, *frxB*, *gyrA*, and *gyrB* of *H. pylori*, each of which were associated with antibiotic resistance. The virulence gene *cagA* was also included in the custom panel design.

### 2.3 Amplicon design for targeting *H. pylori* genes for custom NGS panel for personalized *H. pylori* eradication treatment

The design of the amplicons that target the selected *H. pylori* regions was carried out with an Ion Ampliseq designer (ThermoFisher Scientific, Waltham, MA, USA). The genomic position of each target region referred to the position in *Helicobacter pylori* strain 26695 (GenBank (Benson et al., 2018): NC_018939).

Independent primer sets have been designed for *HPrrna23S* and *HPrrna16S* to target the regions associated with antibiotic resistance to clarithromycin and tetracycline based on the genome sequence of *H. pylori* strain 26695 (**Supplementary Table 1**). Two primer sets, P1 and P2, were designed to cover target regions of *HP23rrna*. Positions 965–967 of *HPrrna16S* were covered by another two primer sets, P3 and P4. P1 and P3 were grouped together as amplicon pool 3, and P2 and P4 as amplicon pool 4 for inhibiting primer interference.

Consequently, NGS-PHET consists of five amplicon pools targeting multiple regions of the genomes of the host and *H. pylori*. Pools 1 and 2 contain amplicons targeting seven genes including *pbp1*, *rdxA*, *frxA*, *frxB*, *gyrA*, *gyrB*, and *cagA*, and pools 3 and 4 contain amplicons targeting *HPrrna23S* and *HPrrna16S*. Pool 5 contains amplicons targeting the whole exome of the host.

### 2.4 Customized NGS library preparation method for the two species and sequencing

Simultaneous DNA extraction for genomic DNA of *H. pylori* and the human host was performed directly from the gastric biopsy without culture using a QIAamp DNA mini kit (QIAGEN, Hilden, Germany).

NGS libraries were constructed using an Ion AmpliSeq library kit plus (ThermoFisher Scientific, Waltham, MA, USA). Amplification master mix was prepared using 5X Ion AmpliSeq HiFi mix and total genomic DNA in adherence to the manufacturer’s instructions. For pools 1 and 2, target region amplification was performed by adding 10 µL of 2X Ion AmpliSeq primer pool to the master mix containing 14.3 ng of genomic DNA under the following conditions: 99°C for 2 minutes, followed by 19 cycles at 99°C for 15 seconds, and 60°C for 4 minutes. Pools 3 and 4 were amplified by running the following program after adding 0.6 mM of primers to the master mix containing 14.3 ng of genomic DNA: 99°C for 2 minutes, followed by 21 cycles at 99°C for 15 seconds, and 60°C for 4 minutes. The master mix containing 68.6 ng of genomic DNA was used for the human whole exome library construction using an Ion AmpliSeq exome RDY kit plus (ThermoFisher Scientific, Waltham, MA, USA) and the amplification reaction followed the manufacturer’s instructions.

After target region amplification, pools 1–4 were combined in a single well, and the human whole exome libraries were also transferred to another single well. The primer sequences were digested to perform adapter sequence ligation and barcoded using an Ion Xpress barcode adapters kit (ThermoFisher Scientific, Waltham, MA, USA). The libraries of pools 1–4 and the human whole exome libraries were attached with different barcodes for identification. The barcoded libraries were purified using AMPure XP reagent (Beckman Coulter, Inc., Brea, CA, USA) and global amplification was performed. The libraries were quantified to 100pmol using an Agilent 2100 bioanalyzer (Agilent, Santa Clara, CA, USA) after being purified two times with AMPure XP reagent.

The quantified 5 µL of the libraries of pools 1–4 were combined with 15 µL of the quantified human whole exome libraries for templating. The combined libraries were loaded onto an Ion chef instrument and template preparation was performed using an Ion 540 kit-chef (ThermoFisher Scientific, Waltham, MA, USA). After the template was loaded on an Ion 540 chip, the chip was transferred to Ion S5XL sequencing systems and sequencing was performed using Ion S5 sequencing solutions and Ion S5 sequencing reagents (ThermoFisher Scientific, Waltham, MA, USA) following the manufacturer’s instructions.

### 2.5 Bioinformatics pipeline

A customized reference file that combined human reference genome build 19 (hg19) and *H. pylori* 26695 was generated to align the target region sequence of NGS-PHET. The target region information of pools 3 and 4 was added to the BED file that contains the information of pools 1 and 2, which was produced using the Ion Ampliseq designer. The generated new BED file was applied for the read alignment. The Torrent mapping alignment program (TMAP) (version 4.4, Torrent Suite Software, ThermoFisher Scientific, Waltham, MA, US) and Ion Torrent variant caller (Torrent Suite Software, ThermoFisher Scientific, Waltham, MA, US) were used to process the raw data into variant call format (VCF) files with default parameters of germline low stringency mode. Reads were mapped to the hg19 or *H. pylori* 26695, and the low-quality bases with Phred quality scores below 20 were filtered out. The cutoff values were as follows: allele frequency, >0.18 (SNV) and >0.23 (indel); coverage, >35 (SNV) and >40 (indel); coverage on either strand, >3 (SNV) and >3 (indel); strand bias, <0.95 (SNV) and <0.75 (indel); relative read quality, >6.5 (SNV); common signal shift, <0.25; reference/variant signal shift, <0.2 (insertion); and reference/variant signal, shift <0.2 (deletions). Reads from the S5XL sequencer are heterogeneous in length, and any additional filtering or trimming steps were not applied.

For WES data, the process of generated reads to VCF files was performed by using the TMAP and Ion Torrent variant caller with the default parameters of the germline high stringency setting following the manufacturer’s instructions. Reads were mapped to hg19 and low-quality bases with Phred quality scores below 20 were filtered out.

### 2.6 Proposed criteria for interpretation of sequence variants and personalized treatment suggestions

The variants identified within the *H. pylori* genome were interpreted with reference to previous studies for the purpose of determining resistance to each antibiotic (**Table 1**). An *H. pylori* strain containing any of the missense mutations occurring in Phe366, Gly367, Ala369, Ser414, Val469, Phe473, Thr541, Ser543, Thr556, Thr558, Asn560, or Asn562 of Pbp1 was classified as exhibiting amoxicillin resistance (Okamoto et al., 2002; Kwon et al., 2003; Gerrits et al., 2006; Rimbara et al., 2007; Rimbara et al, 2008; Qureshi et al., 2011; Zerbetto De Palma et al., 2017; Kuo et al., 2023). Clarithromycin resistance of an *H. pylori* strain was determined through the identification of A2142G/C, A2143G, or A2144G in *HPrrna23S* (Nishizawa and Suzuki, 2014). The presence of nonsense or frameshift mutations causing amino acid truncation, or missense mutations occurring in Arg16, His17, Ser18, Lys20, Arg41, Leu42, Ser43, Ser45, Ser46, Tyr47, Asn48, Gln50, Val55, Met56, Asn73, Ile142, Gly145, Gly149, Ile160, Gly162, Gly163, Lys200, Lys202, or Leu209 of RdxA in an *H. pylori* strain was the criterion for classifying it as a metronidazole-resistance strain (Martínez-Júlvez et al., 2012). If nonsense or frameshift mutations causing amino acid truncation of FrxA or FrxB were concomitant with the RdxA mutations described above in one strain, the strain was categorized as exhibiting strong metronidazole resistance (Kwon et al., 2000; Nishizawa and Suzuki, 2014). The other remaining variants observed in *frxA* or *frxB* were not possible to evaluate for the induction of inactivation; hence, these variants were not considered for resistance classification. Tetracycline resistance classification in an *H. pylori* strain was established through the identification of AGA926–928→TTC in *HPrrna16S* (Pioletti et al., 2001; Bauer et al., 2004; Chukwudi, 2016). Levofloxacin resistance in an *H. pylori* strain was determined by detecting Asn87Lys/Tyr or Asp91Gly/Asn/Tyr in GyrA, or missense mutations in QRDR of GyrB (Glu415-Ser454) (Moore et al., 1995; Nishizawa et al., 2009; Lee et al., 2011; Nishizawa and Suzuki, 2014; Mori et al., 2016; López-Gasca et al., 2018; Chu et al., 2020). For *cagA*, no read depth identification in any amplicons was classified as negative, while positive was assigned if read counts were observed in the amplicons.

**Table 1:** Antibiotic resistance classification criteria of *H. pylori* using previous reports.

antibiotics		amoxicillin	clarithromycin	metronidazole	tetracycline	levofloxacin
resistance associated residues		Phe366, Gly367, Ala369, Ser414, Val469, Phe473, Thr541, Ser543, Thr556, Thr558, Asn560 and Asn562 of Pbp1	A2142, A2143, and A2144 of *HPrrna23S*	Arg16, His17, Ser18, Lys20, Arg41, Leu42, Ser43, Ser45, Ser46, Tyr47, Asn48, Gln50, Val55, Met56, Asn73, Ile142, Gly145, Met149, Ile160, Gly162, Gly163, Arg200, Ser202, and Trp209 of RdxA, and nonsense mutation, frameshift indel causing premature termination of RdxA, FrxA, and FrxB	AGA926–928TTC of *HPrrna16S*	Asn87Lys/Tyr and Asp91Gly/Asn/Tyr of GyrA and quinolones resistance-determining region of GyrB (Glu415-Ser454)
resistance classification criteria	susceptible	no variant at the positions	no variant at the positions	no variant at the positions	no variant at the positions	no variant at the positions
	resistance	harboring at least one of the variants	harboring at least one of the variants	harboring at least one of the variants	harboring at least one of the variants	harboring at least one of the variants in gyrA and/or gyrB
	strong resistance	N/A	N/A	harboring at least one of the variants in *frxA* or *frxB* with *rdxA*	N/A	N/A

N/A, not applicable.

**Figure 2:**
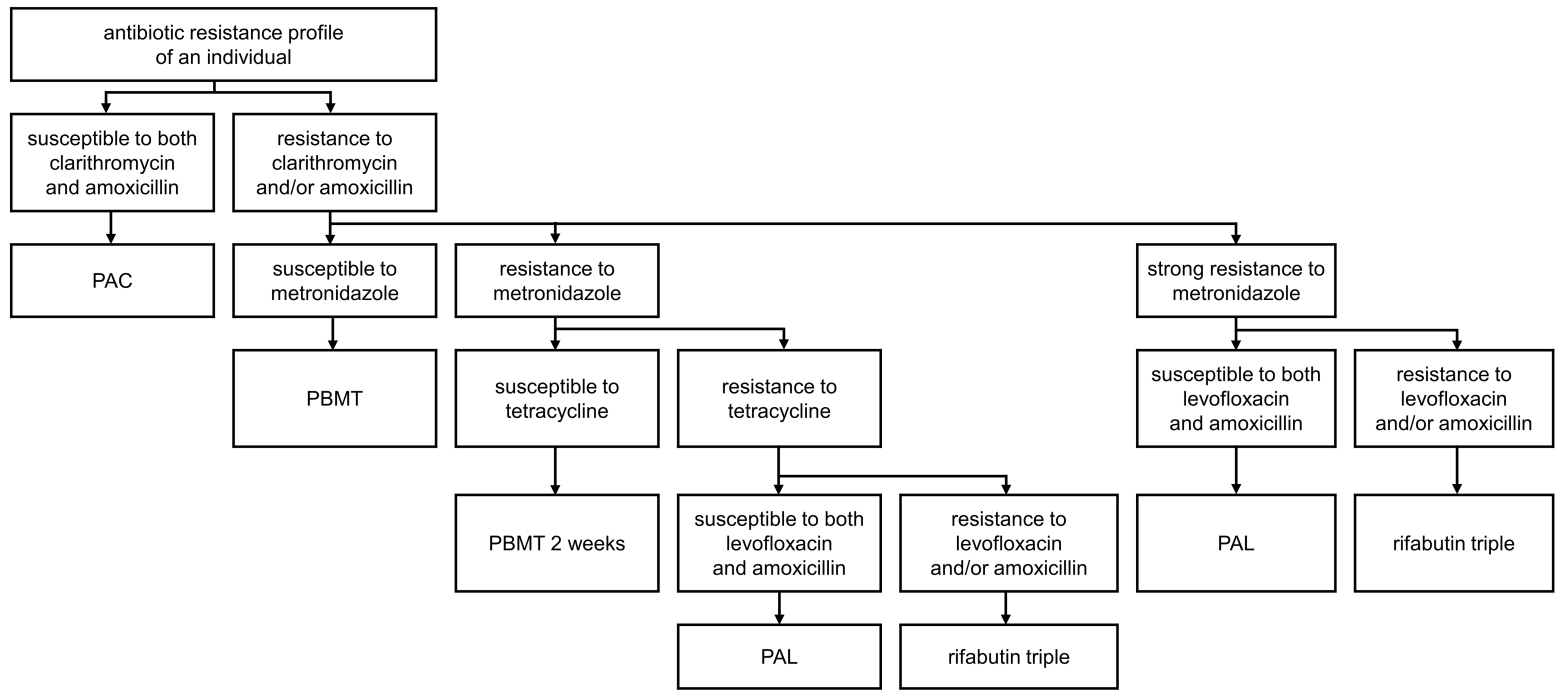
The customized framework criteria for personalized treatment suggestions based on the antibiotic resistance profile of each subject. A personalized treatment for each subject was suggested according to the customized framework criteria, which were designed based on the algorithm for empirical *H. pylori* eradication presented in the Maastricht VI/Florence Consensus report with the antibiotics used in SMG-SNU Boramae Medical Center. PAC, proton pump inhibitor (lansoprazole), amoxicillin, and clarithromycin; PBMT, proton pump inhibitor (lansoprazole), bismuth, metronidazole, and tetracycline; PAL, proton pump inhibitor (lansoprazole), amoxicillin, and levofloxacin.

Personalized treatment suggestion was performed according to customized framework criteria with the antibiotic resistance profile of each subject derived from the variant interpretation result. The customized framework criteria were designed based on the algorithm for empirical *H. pylori* eradication presented in the Maastricht VI/Florence Consensus report, considering previous studies and the antibiotics used at the SMG-SNU Boramae Medical Center (**Figure 2**) (Malfertheiner et al., 2022). The eradication rate of PAC treatment in clarithromycin-resistant strains was approximately 65% and that of amoxicillin-resistant strains was approximately 75%, both below 80%, the acceptable first-line *H. pylori* eradication therapy success rate (Graham and Fischbach, 2010; Hwang et al., 2010; Kim et al., 2015; Chen et al., 2017). Thus, we suggest PAC if an *H. pylori* strain is susceptible to both clarithromycin and amoxicillin. For the strains resistant to either clarithromycin or amoxicillin, PBMT is recommended if the strain is susceptible to metronidazole, and 2-week PBMT treatment is suggested if the strain is resistant to metronidazole (Lee et al., 2010). If a strain is resistant to both metronidazole and tetracycline or exhibits strong resistance to metronidazole, PAL is suggested. At this stage, a rifabutin triple therapy is recommended for a strain resistant to either levofloxacin or amoxicillin. The proposed treatment for each subject was validated with the treatment that each subject achieved *H. pylori* eradication with.

For human WES data, star alleles of *CYP3A4* were extracted for each subject that matched the allele definition table of *CYP3A4* sourced from the Pharmacogene Variation Consortium (PharmVar) released in August 2021 (Gaedigk et al., 2021) and translated into the CYP3A4 phenotype grounded in the Royal Dutch Pharmacists Association for the advancement of the Dutch Pharmacogenetics Working Group’s (DPWG) phenotype groups for *CYP3A4* provided by the Pharmacogenomics Knowledge Base (PharmGKB) (Whirl-Carrillo et al., 2012). Star alleles of *CYP2C19* were obtained for each subject, matching the allele definition table of *CYP2C19* sourced from PharmVar that was released in August 2021 and translated into the CYP2C19 phenotype on the diplotype-phenotype table of *CYP2C19* provided by PharmGKB. Subjects were assigned to homozygous EM, heterozygous EM, and PM.

## 3 Results

### 3.1 Simultaneous genomic DNA extraction of the two species

Genomic DNA was extracted directly from gastric biopsies of 12 subjects, who satisfied the inclusion criteria, and four controls: three positive controls and one negative control. All participants were Koreans and the clinical data of each subject were obtained. The positive controls and negative control were registered to verify the effectiveness of NGS-PHET. The PCR results of the controls confirmed that the primers of pools 3 and 4 effectively amplified the target regions without any non-specific bindings, while human glyceraldehyde-3-phosphate dehydrogenase (h*GAPDH*) was fully amplified as a positive control (**Supplementary Figure 1**).

### 3.2 Sequencing performance of custom NGS panel

For *pbp1*, *rdxA*, *frxA*, *frxB*, *gyrA*, *gyrB*, and *cagA*, the amplicons designed to cover 100.0% of the target region were separated into two amplicon pools; pool 1 consisted of 36 amplicons and pool 2 consisted of 37 amplicons (**Supplementary Table 2**). The range in the size of the amplicons was from 125 to 275 bps and the total target area covered by the two pools was 13.8 kbps. It was impossible to construct amplicons using Ion Ampliseq designer for *HPrrna23S* and *HPrrna16S*, thus amplicons were designed manually and separated into two amplicon pools. Pool 3 consisted of two amplicons and pool 4 consisted of two amplicons and was designed to cover 100.0% of the target region. The range in size of the amplicons was from 301 to 405 bps and the total target area covered by the two pools was 2.0 kbps. For the human exome, the Ion Ampliseq exome panel, which is a commercial NGS panel covering >97% of the human exome, was used as the amplicon pool 5 of NGS-PHET. The range in size of the amplicons was from 200 to 350 bps and the total target area covered by the pool was 33 Mbps.

**Figure 3:**
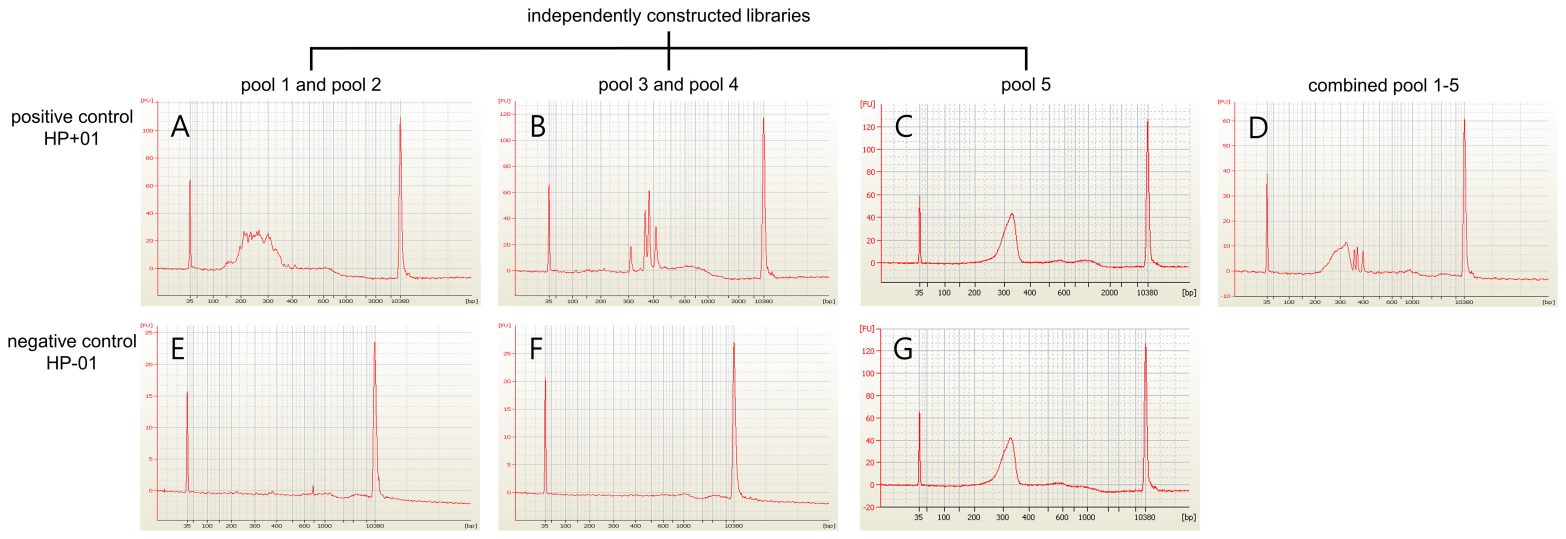
NGS libraries quantifies the results of the positive control and negative control. The NGS libraries’ construction using the positive control (HP+01) and the negative control (HP-01) were quantified using a bioanalyzer. **(A-D)** The positive control identified the appropriate quantity of NGS libraries. **(E-G)** The libraries targeting the *H. pylori* genome were not constructed in the negative control while the human whole exome library was constructed successfully.

**Figure 4:**
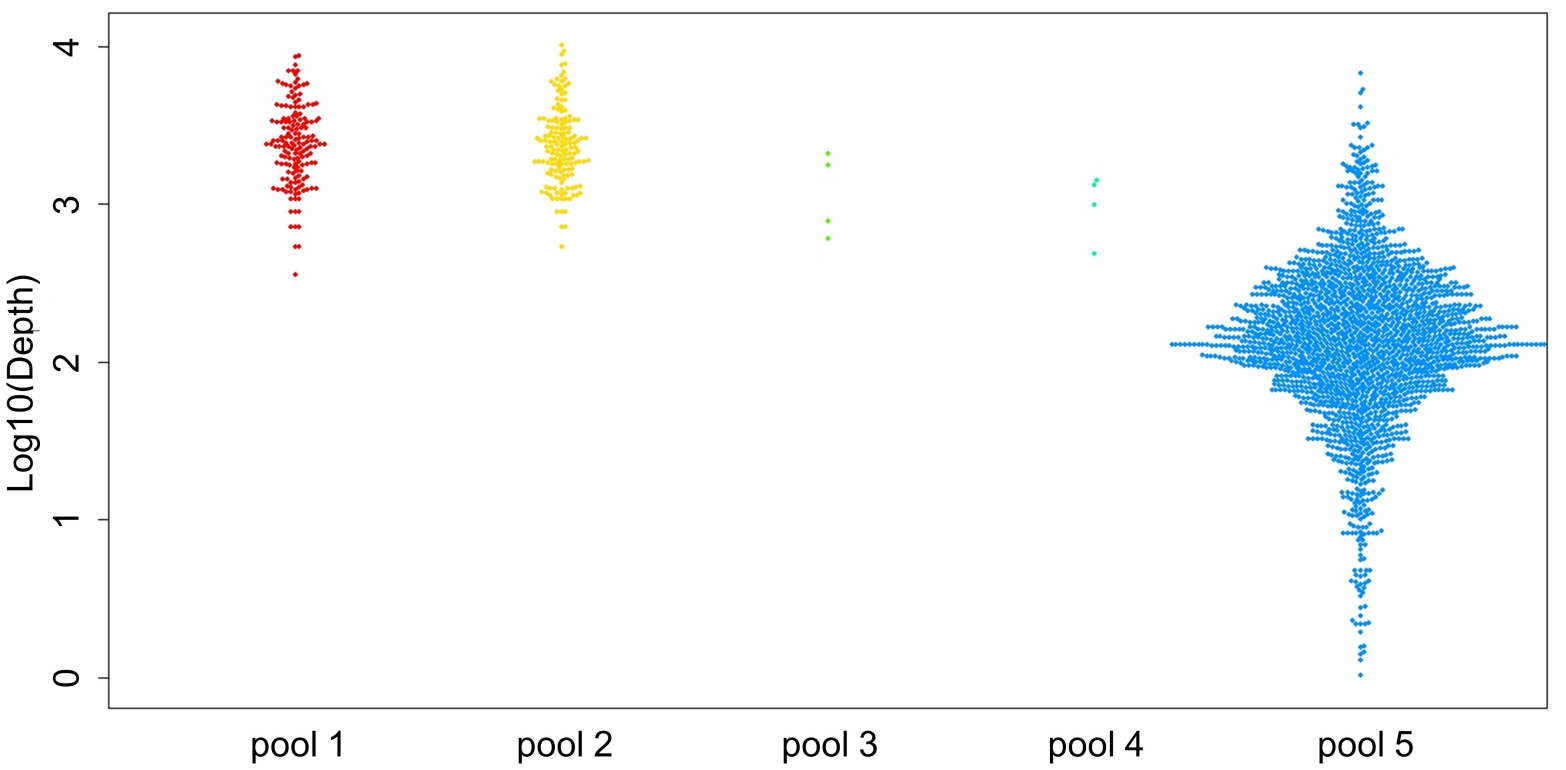
Sequencing depth results of the positive controls. The mean depth of each amplicon is displayed as a single dot using the three positive controls. Each pool is indicated as a dot with the following colors: pool 1 in red, pool 2 in yellow, pool 3 in light green, pool 4 in green, and pool 5 in blue.

Three positive control samples were registered to modify the sequencing read depth for NGS-PHET. As quantifying *H. pylori* DNA was impossible since genomic DNA was extracted without species separation, different amplification conditions were assigned to each of the five pools constituting NGS-PHET to obtain a substantial *H. pylori* DNA sequence read depth. NGS library construction was confirmed through the bioanalyzer results and the library construction of *H. pylori* target regions was not observed in the bioanalyzer results of the negative control (**Figure 3**). The reads obtained from the simultaneous sequencing of the NGS-PHET library of the positive control, each barcoded with distinct barcodes, were aligned with the combined BED file. Through coverage analysis, it was verified that *H. pylori* and human sequences were not intermixed in their respective libraries and existed independently (**Supplementary Table 3**). For the positive controls, the individual sequencing depth of each target gene was not analyzed and derived, and the sequencing depth was reviewed only for six randomly selected genes including *HPrrna23S*, *HPrrna16S*, *rdxA*, *gyrA*, *CYP3A4*, and *CYP2C19* to ensure that DNA amplification was done appropriately for data analysis. Based on the results of NGS for the positive control samples, the average depth was 2081 for pools 1 and 2, 2000 for pool 3, 800 for pool 4, and 124 for pool 5, respectively (**Figure 4**). The efficient read depth numbers for host DNA and *H. pylori* DNA were sufficient for subsequent analyses of SNVs and indels, and the experimental conditions modified to derive these experimental results were applied to all samples registered in this study.

The average of total reads of the pool 1–4 of NGS-PHET target region for the 12 subjects was 106,568,438 (3,866,298–309,207,459). The average depth for the target regions of pools 1 and 2 was 7,722, and for pools 3 and 4 it was 1,315. The average total reads of pool 5 of the 12 subjects was 36,695,844 (21,694,538–71,037,591). The mean depth of the whole exome panel was 115 and the average depth of the target genes, *CYP2C19* and *CYP3A4*, was 100.

### 3.3 Sequencing results of the customized NGS panel

As a result of performing simultaneous sequencing for multiple species including the host and *H. pylori* by using NGS-PHET, VCF files containing host variant information and *H. pylori* variant information were obtained for each patient’s DNA sample and *H. pylori* variants affecting antibiotic resistance and host variants affecting PPI metabolism were concurrently identified without additional species-specific analysis (**Tables 2**, **3**).

**Table 2:** Star allele genotype and phenotype of proton pump inhibitor metabolism-associated genes.

subject	*CYP2C19*		*CYP3A4*	
	star allele genotype	phenotype	star allele genotype	phenotype
HP01	*1|*2	heterozygous extensive metabolizer	*1|*1	normal metabolizer
HP02	*1|*1	homozygous extensive metabolizer	*1|*1	normal metabolizer
HP03	*1|*35	heterozygous extensive metabolizer	*1|*1	normal metabolizer
HP04	*2|*2	poor metabolizer	*1|*1	normal metabolizer
HP05	*1|*1	homozygous extensive metabolizer	*1|*18	intermediate metabolizer
HP06	*1|*1	homozygous extensive metabolizer	*1|*18	intermediate metabolizer
HP07	*1|*2	heterozygous extensive metabolizer	*1|*1	normal metabolizer
HP08	*1|*1	homozygous extensive metabolizer	*1|*1	normal metabolizer
HP09	*1|*2	heterozygous extensive metabolizer	*1|*1	normal metabolizer
HP10	*1|*1	homozygous extensive metabolizer	*1|*1	normal metabolizer
HP11	*1|*2	heterozygous extensive metabolizer	*1|*18	intermediate metabolizer
HP12	*1|*2	heterozygous extensive metabolizer	*1|*1	normal metabolizer

**Table 3:** Identifying the variants associated with antibiotic resistance.

gene	*HPrna23S*	*HPrna16S*	*pbp1*	*rdxA*	*frxA*	*frxB*	*gyrA*	*gyrB*	*cagA*
associated antibiotics	clarithromycin	tetracycline	amoxicillin	metronidazole			levofloxacin		-
HP01	N.D.	N.D.	N.D.	N.D.	Leu33Val fs*2	Lys242Asn fs*8	N.D.	N.D.	positive
HP02	A2143G	N.D.	N.D.	N.D.	N.D.	N.D.	N.D.	N.D.	positive
HP03	A2143G	N.D.	N.D.	N.D.	Ala15Arg fs*7	N.D.	N.D.	N.D.	positive
HP04	A2143G	N.D.	Asn562Tyr	N.D.	Asn56Met fs*3	N.D.	N.D.	N.D.	positive
HP05	N.D.	N.D.	N.D.	N.D.	N.D.	N.D.	N.D.	N.D.	positive
HP06	N.D.	N.D.	Asn562Lys	N.D.	N.D.	N.D.	N.D.	N.D.	positive
HP07	N.D.	N.D.	N.D.	Arg16His	N.D.	N.D.	N.D.	N.D.	positive
HP08	A2143G	AGA926–928TGA	N.D.	Gln50*	N.D.	Phe32Leu fs*10	Asn87Lys	N.D.	positive
HP09	A2143G	N.D.	N.D.	N.D.	N.D.	Phe32Leu fs*10	N.D.	N.D.	positive
HP10	N.D.	N.D.	N.D.	N.D.	N.D.	N.D.	N.D.	N.D.	positive
HP11	A2143G	AGA926–928TGA	N.D.	N.D.	Ala15Arg fs*7	N.D.	N.D.	N.D.	positive
HP12	N.D.	N.D.	N.D.	N.D.	Asn56Met fs*3	Arg132Pro fs*1	N.D.	N.D.	positive

N.D., not detected.

For host variants affecting PPI metabolism, the metabolic phenotype of each patient was classified grounded in star alleles of *CYP3A4* and *CYP2C19*. By matching the star alleles of *CYP3A4*, HP05, HP06, and HP11 were determined to be intermediate metabolizers (*1|*18), while all other subjects were determined to be normal metabolizers (*1|*1). The phenotype assignment results of *CYP2C19* revealed five homozygous Ems: HP02 (*1|*1), HP05 (*1|*1), HP06 (*1|*1), HP08 (*1|*1), and HP10 (*1|*1); six heterozygous Ems: HP01 (*1|*2), HP03 (*1|*35), HP07 (*1|*2), HP09 (*1|*2), HP11 (*1|*2), and HP12 (*1|*2); and one PM, HP04 (*2|*2).

For *H. pylori* variants affecting antibiotic resistance, A2143G in *HPrna23S* was indicated in six subjects (HP02, HP03, HP04, HP08, HP09, and HP11) and no other variants were found in *HPrna23S* in the remaining six subjects. AGA926–928TTC of *HPrrna16S* was not identified among the 12 subjects, but a novel nonsense mutation A926T was indicated in HP08 and HP11. For *pbp1*, Asn562Tyr was detected in HP04 and Asn562Lys was revealed in HP06. In *rdxA*, Arg16His was indicated in HP07 and Gln50* was identified in HP08. Within *frxA*, HP01 (Leu33Valfs*2), HP03 (Ala15Arg fs*7), HP04 (Asn56Metfs*3), HP11 (Ala15Arg fs*7), and HP12 (Asn56Metfs*3) each had one frameshift truncation mutation. A single frameshift truncation mutation in *frxB* was identified for HP01 (Lys242Asnfs*8), HP08 (Phe32Leu fs*10), HP09 (Phe32Leu fs*10), and HP12 (Arg132Pro fs*1). Asn87Lys was indicated in *gyrA* in HP08, and no other variants were found in *gyrA* and *gyrB* in all other subjects. *cagA* was positive in all subjects.

### 3.4 Interpretation results and validation

The patients enrolled in this study provided gastric biopsy samples for genetic variant analysis before the start of eradication treatment, and then received eradication treatment in clinical practice according to the general *H. pylori* eradication treatment method performed in Korea. After the patient’s treatment was completed, the data on the results of eradication treatment outcomes verified by professional clinicians were reviewed for consistency with the proposed personalized *H. pylori* eradication treatment method according to the customized framework criteria based on the NGS-PHET results.

HP05, HP06, and HP11 were assigned to decrease the function allele CYP3A4*18. However, since metabolic reduction of PPI by CYP3A4*18 was not reported, it was considered to have no effect (**Table 2**). For *CYP2C19*, HP02, HP05, HP06, HP08, and HP10 were classified as homozygous EM (**Table 2**). Thus, it was recommended that these six subjects increase their PPI dose to adequately control gastric pH based on existing guidelines.

The *H. pylori* strains affecting HP04 and HP06 were categorized as amoxicillin-resistant because of the alterations at Asn562 (**Table 4**). The *H. pylori* in HP02, HP03, HP04, HP08, HP09, and HP11 were classified as clarithromycin-resistant strains due to A2143G in HPrna23S. The presence of Arg16His in rdxA was the reason for classifying HP07 as exhibiting metronidazole resistance. The inactivation of RdxA and FrxB were the basis for categorizing HP08 as demonstrating strong resistance to metronidazole. Tetracycline-resistant associated mutations were not identified in any of the 12 samples. Asn87Lys was identified in GyrA of HP08, which was consequently classified as representing levofloxacin resistance.

**Table 4:** Antibiotic resistance profile of each subject and suggested treatment.

subject	antibiotic resistance profile					treatment suggestion	treatment with confirmed *H. pylori* eradication achievement
	amoxicillin	clarithromycin	levofloxacin	metronidazole	tetracycline		
HP01	susceptible	susceptible	susceptible	susceptible	susceptible	PAC	PAC
HP02	susceptible	resistance	susceptible	susceptible	susceptible	PBMT	PBMT
HP03	susceptible	resistance	susceptible	susceptible	susceptible	PBMT	PBMT
HP04	resistance	resistance	susceptible	susceptible	susceptible	PBMT	PBMT
HP05	susceptible	susceptible	susceptible	susceptible	susceptible	PAC	PAC
HP06	resistance	susceptible	susceptible	susceptible	susceptible	PBMT	PBMT
HP07	susceptible	susceptible	susceptible	resistance	susceptible	PAC	PAC
HP08	susceptible	resistance	resistance	resistance	susceptible	rifabutin triple	PBMT
HP09	susceptible	resistance	susceptible	susceptible	susceptible	PBMT	PBMT
HP10	susceptible	susceptible	susceptible	susceptible	susceptible	PAC	PAC
HP11	susceptible	resistance	susceptible	susceptible	susceptible	PBMT	PAL
HP12	susceptible	susceptible	susceptible	susceptible	susceptible	PAC	PBMT

PAC, proton pump inhibitor (lansoprazole), amoxicillin, and clarithromycin; PBMT, proton pump inhibitor (lansoprazole), bismuth, metronidazole, and tetracycline; PAL, proton pump inhibitor (lansoprazole), amoxicillin, and levofloxacin.

The *H. pylori* strains to which HP01, HP05, HP10, and HP12 were exposed were found to be susceptible to all antibiotics; hence, the recommendation for PAC was made according to the customized framework criteria (**Table 4**). Since HP02, HP03, HP09, and HP11 were identified to be carrying clarithromycin-resistant *H. pylori* strains, PBMT was proposed. For HP04, the suggestion following the criteria was PBMT due to the *H. pylori* in HP04 being resistant to amoxicillin and clarithromycin. It was determined that the *H. pylori* strains infecting HP06 were amoxicillin-resistant, thus PBMT was suggested for HP06. The *H. pylori* in HP07 was determined to exhibit metronidazole resistance. In consequence, PAC was proposed for HP07. As HP08 was identified to carry *H. pylori* strains resistant to clarithromycin, levofloxacin, and metronidazole, rifabutin triple treatment was recommended in accordance with the customized framework criteria.

We validated whether each of the proposed treatments based on the customized framework was consistent with the treatment that each subject achieved *H. pylori* eradication with (**Table 4**). For nine of 12 patients, each of the treatments proposed on the basis of the customized framework matched the treatment with which *H. pylori* eradication in each patient was achieved, and not for the remaining three patients.

## 4 Discussion

In the present study, the individual antibiotic resistance profile, derived from the sequencing results of NGS-PHET, was applied to propose personalized *H. pylori* eradication treatments within the customized framework criteria. NGS-PHET can provide genetic information of *H. pylori* genes, including *HPrrna23S*, *HPrrna16S*, *pbp1*, *rdxA*, *frxA*, *frxB*, *gyrA*, *gyrB*, and *cagA*, and the human exome using the DNA of the two species that is extracted simultaneously without culture (**Table 3**). The experimental and analytical procedures developed and modified for this study enable variant identification without species separation by amplifying DNA from the two species at different rates using a relatively small amount of total genomic DNA, and applying appropriate parameters to the data of each species. Additionally, the entire experimental procedure takes less than 48 hours, and a treatment suggestion is provided within 4 days from biopsy, allowing for a relatively rapid test compared to the 2 weeks required for DNA extraction after *H. pylori* isolation (Hulten et al., 2021). This approach is also expected to obtain potential candidates for antibiotic resistance-related variants and promptly detect newly reported resistance-associated mutations within the targeted region by covering a broader region compared to the currently utilized PCR-based tests.

To determine an individual antibiotic resistance profile, experimental data obtained using NGS were analyzed and *H. pylori* mutations were detected (**Table 3**). For *pbp1*, applying PCR assays has been reported to have limitations (Nishizawa and Suzuki, 2014), whereas in this study, by applying NGS-PHET, multiple mutations were detected, and these mutations were found to cause alternation in amoxicillin-resistance-associated residue Asn562. In addition, for metronidazole-resistance-associated genes, which require analysis of the nucleotide sequence of the entire gene to determine the antibiotic resistance, 10 novel truncation mutations inducing protein inactivation were detected. Therefore, by applying the methodology presented in this study to genes in which multiple mutations associated with antibiotic resistance are detected over a wide range, not only can previously reported mutations be accurately identified, but novel mutations can be rapidly detected and presented as antibiotic resistance candidates.

The personalized *H. pylori* eradication treatments were proposed according to the customized framework criteria, and the proposals were validated through the actual treatment that each subject achieved eradication with (**Table 4**). The suggested treatments agreed with the treatments that were prescribed to each subject and achieved eradication in nine of the 12 subjects. Analysis of the first-line treatment outcomes of the 12 subjects revealed that four achieved *H. pylori* eradication, while the remaining eight required second-line treatment. Had the personalized treatments been administered initially, more patients would likely have achieved *H. pylori* eradication with a single treatment. Consequently, the treatment recommended within the customized framework criteria, based on the mutations identified through NGS-PHET, has been indicated to be effective in *H. pylori* eradication and holds the potential to shorten the duration to achieve eradication. In the case of HP08, PAL was recommended because mutations associated with clarithromycin, metronidazole, and levofloxacin resistance were detected, but in actual eradication treatment for the patient, *H. pylori* eradication was confirmed after PBMT. Metronidazole resistance occurs due to RdxA inactivation, and increases with further inactivation of FrxA and/or FrxB (Nishizawa and Suzuki, 2014). In HP08, inactivation-related mutations of both *rdxA* and *frxB* were detected and found to be resistant to metronidazole, but the eradication was considered to have been successful due to PBMT prescription containing a sufficient dose of bismuth. Since clarithromycin resistance-related mutation were detected in HP11, PBMT was proposed, but in actual treatment, eradication was achieved through PAL. In addition, although any known mutations related to clarithromycin or amoxicillin resistance were not detected in HP12, *H. pylori* eradication was achieved through PBMT in actual eradication treatment. For these two subjects, it was considered that resistance-related mutations that have not yet been reported are the cause, and the use of molecular tests targeting a wide range is essential to identify the cause and treat patients.

The limitation of this study is that it is impossible to quantify *H. pylori* DNA because genomic DNA was extracted from gastric biopsy without species separation, and it is difficult to uniformly amplify the entire target region. To solve this problem, the amplification amount was improved by diversifying the amplification cycle of pools 1–4, but it was nevertheless observed that sequencing depth decreased in the *cagA* regions of HP01, HP02, and HP06. It was considered that this can be solved by adding a primer for specific area. In addition, although more *H. pylori* candidate genes have been secured due to a lack of information on the genes and variants, they have not been added to the target area at present. In the future, when scientific evidence for the areas is secured, it will be added to the panel.

In summary, a custom NGS panel for personalized *H. pylori* eradication treatment (NGS-PHET), which is a multi-species integrated NGS panel that targets antibiotic resistance associated *H. pylori* genes and host genes involved in PPI metabolism, was designed and an experimental procedure was developed. Identified variants were interpreted using the customized framework criteria based on previously reported variants associated with antibiotic resistance of *H. pylori* and PPI metabolism of the host and the individualized *H. pylori* eradication treatment was suggested. NGS-PHET is a more efficient and cost-effective approach compared to whole genome sequencing, and since DNA is extracted directly from gastric biopsy, the time required for the examination can be significantly reduced compared to the culture-based method. Furthermore, the genetic information of the host and *H. pylori* related to eradication treatment is simultaneously detected, and the type and amount of drugs to be administered to the patients are determined through a customized framework. By applying the methodology using NGS-PHET and the custom framework, antibiotic-resistance mutations are accurately detected and a customized eradication treatment is proposed, which not only helps the eradication treatment perform quickly and effectively in most patients with antibiotic-resistant *H. pylori* strains, but can also be used in research to find novel candidates for antibiotic-resistance mutations.

## Data Availability

The datasets presented in this study can be found in online repository. The names of the repository and accession number can be found below: https://www.ebi.ac.uk/eva/?eva-study=PRJEB79130.
