# Corrigendum: Development and validation of next-generation sequencing panel for personalized *Helicobacter pylori* eradication treatment targeting multiple species

**DOI:** 10.3389/fcimb.2024.1502072

**Published:** 2024-10-25

**Authors:** Byung-Joo Min, Myung-Eui Seo, Jung Ho Bae, Ji Won Kim, Ju Han Kim

**Affiliations:** ^1^ Forensic DNA Division, National Forensic Service Seoul Institute, Seoul, Republic of Korea; ^2^ Seoul National University Biomedical Informatics (SNUBI), Department of Biomedical Sciences, Seoul National University College of Medicine, Seoul, Republic of Korea; ^3^ Department of Internal Medicine and Healthcare Research Institute, Healthcare System Gangnam Center, Seoul National University Hospital, Seoul, Republic of Korea; ^4^ Department of Internal Medicine, Seoul Metropolitan Government - Seoul National University Boramae Medical Center, Seoul, Republic of Korea; ^5^ Department of Internal Medicine, Seoul National University College of Medicine, Seoul, Republic of Korea; ^6^ Seoul National University Biomedical Informatics (SNUBI), Division of Biomedical Informatics, Seoul National University College of Medicine, Seoul, Republic of Korea

**Keywords:** personalized *Helicobacter pylori* eradication treatment, individual antibiotic resistance profile, antibiotic resistance, proton-pump inhibitor metabolic phenotype, multispecies integrated next-generation sequencing panel

In the published article, there was an error in the **Funding** statement. The **Funding** statement for the National Research Foundation of Korea (NRF) grant funded by the Korea government (Ministry of Science and ICT) was displayed as “NRF-2023R1A2C300768”. The correct **Funding** statement appears below.

“The author(s) declare financial support was received for the research, authorship, and/or publication of this article. This research was supported by a grant from Seoul national university Hospital R&D Foundation in the Republic of Korea, Collaborative Research Program of SNU Boramae Medical Center and Basic Medical Science from Seoul National University College of Medicine, and the National Research Foundation of Korea (NRF) grant funded by the Korea government (Ministry of Science and ICT). Funder: Seoul national university Hospital R&D Foundation, Award Number: 800–20220003, Grant Recipient: JHK. Funder: SNU Boramae Medical Center and Basic Medical Science from Seoul National University College of Medicine, Award Number: 800–20220002, Grant Recipient: JWK. Funder: National Research Foundation of Korea (NRF) grant funded by the Korea government (Ministry of Science and ICT), Award Number: NRF-2023R1A2C3007686, Grant Recipient: JHK.”

The authors apologize for this error and state that this does not change the scientific conclusions of the article in any way. The original article has been updated.

