# Supplementary material for: Development and validation of next-generation sequencing panel for personalized Helicobacter pylori eradication treatment targeting multiple species
Source: Front Cell Infect Microbiol. 2024 Aug 29;14:1379790. doi: 10.3389/fcimb.2024.1379790 (PMC11390507; doi:10.3389/fcimb.2024.1379790)
Supplement: Supplementary file 1 [file DataSheet1.docx]

Supplementary Material

# Supplementary Figures and Tables

## Supplementary Figures


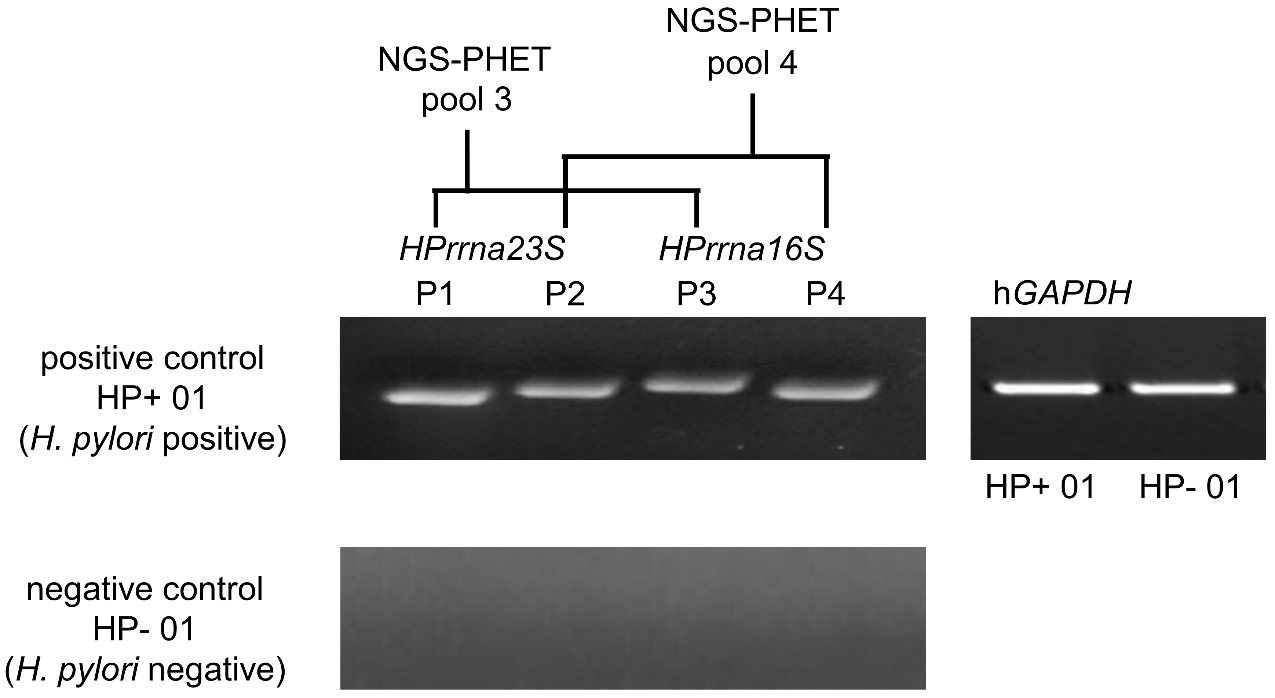


**Supplementary Figure 1.** Target region amplicon detection of the pool 2 and pool 3 through PCR using positive control and negative control. The total genomic DNA of positive control (HP+01) and negative control (HP-01) were used for amplifying *HPrrna23S* and *HPrrna16S* regions. Primer set P1 and P2 were targeting *HPrrna23S* and primer set P3 and P4 were targeting *HPrrna16S*. The amplicon size of each primer set was as follows; P1 301 bp, P2 361 bp, P3 405 bp, and P4 381bp. There was no other detectable band on the wells where the PCR products were loaded.

## Supplementary Tables

**Supplementary Table 1.** Primer sets for *HPrrna23S* and *HPrrna16S* amplification.

| species | gene | forward primer (5' to 3') | reverse primer (5' to 3') | product size (bp) | primer set name |
| --- | --- | --- | --- | --- | --- |
| *H. pylori* | *HPrrna23S* | CTGCATGAATGGCGTAACGAG | GAGCGACCGCCCCGATCAAAC | 301 | P1 |
|  | *HPrrna23S* | ATGAATGGCGTAACGAGATG | ACACTCAACTTGCGATTTCC | 361 | P2 |
|  | *HPrrna16S* | AGAGTTTGATCCTGGCTCAG | ATCCTAAAACCTTCATCCTC | 405 | P3 |
|  | *HPrrna16S* | CGACCTGCTGGAACATT | TCGTTGCGGGACTTAACCCAA | 381 | P4 |
| human | h*GAPDH* | GCCTTCCGTGTCCCCACT | TGAGGGGGCCCTCCGACG | 118 | h*GAPDH* |

**Supplementary table 2.** Amplicon composition of amplicon pool 1 and pool 2

| pool | gene | amplicon start position at *H. pylori* strain 26695 | amplicon end position at *H. pylori* strain 26695 |
| --- | --- | --- | --- |
| 1 | *gyrB* | 527861 | 528083 |
|  |  | 528214 | 528433 |
|  |  | 528564 | 528752 |
|  |  | 528918 | 529137 |
|  |  | 529261 | 529422 |
|  |  | 529612 | 529821 |
|  | *cagA* | 579849 | 580068 |
|  |  | 580263 | 580437 |
|  |  | 580626 | 580808 |
|  |  | 581002 | 581218 |
|  |  | 581388 | 581496 |
|  |  | 581677 | 581893 |
|  |  | 582089 | 582315 |
|  |  | 582505 | 582681 |
|  |  | 582878 | 583103 |
|  |  | 583261 | 583481 |
|  | *pbp1* | 630981 | 631111 |
|  |  | 631286 | 631509 |
|  |  | 631677 | 631901 |
|  |  | 632067 | 632263 |
|  |  | 632418 | 632638 |
|  | *frxA* | 687793 | 687979 |
|  |  | 688174 | 688387 |
|  | *gyrA* | 752396 | 752620 |
|  |  | 752716 | 752933 |
|  |  | 753126 | 753331 |
|  |  | 753456 | 753666 |
|  |  | 753867 | 754091 |
|  |  | 754289 | 754466 |
|  |  | 754668 | 754850 |
|  | *rdxA* | 1013676 | 1013898 |
|  |  | 1014089 | 1014302 |
|  | *frxB* | 1581432 | 1581631 |
|  |  | 1581790 | 1581990 |
|  |  | 1582183 | 1582320 |
|  |  | 1582514 | 1582708 |
| 2 | *gyrB* | 527646 | 527872 |
|  |  | 528072 | 528225 |
|  |  | 528422 | 528575 |
|  |  | 528741 | 528929 |
|  |  | 529126 | 529272 |
|  |  | 529411 | 529623 |
|  |  | 529810 | 530007 |
|  | *cagA* | 580057 | 580274 |
|  |  | 580426 | 580637 |
|  |  | 580797 | 581013 |
|  |  | 581205 | 581399 |
|  |  | 581485 | 581688 |
|  |  | 581882 | 582100 |
|  |  | 582304 | 582516 |
|  |  | 582670 | 582889 |
|  |  | 583092 | 583272 |
|  | *pbp1* | 630768 | 630992 |
|  |  | 631100 | 631297 |
|  |  | 631498 | 631688 |
|  |  | 631890 | 632078 |
|  |  | 632252 | 632429 |
|  |  | 632627 | 632847 |
|  | *frxA* | 687968 | 688185 |
|  |  | 688376 | 688581 |
|  | *gyrA* | 752609 | 752727 |
|  |  | 752922 | 753137 |
|  |  | 753320 | 753467 |
|  |  | 753655 | 753878 |
|  |  | 754080 | 754300 |
|  |  | 754455 | 754679 |
|  |  | 754839 | 755038 |
|  | *rdxA* | 1013480 | 1013687 |
|  |  | 1013887 | 1014100 |
|  | *frxB* | 1581620 | 1581801 |
|  |  | 1581979 | 1582194 |
|  |  | 1582309 | 1582525 |
|  |  | 1582697 | 1582883 |

**Supplementary table 3.** Average sequencing depth for target regions in positive and negative controls

| species | human | | *H. pylori* | | | |
| --- | --- | --- | --- | --- | --- | --- |
| gene | *CYP2C19* | *CYP3A4* | *HPrrnA23S* | *HPrrnA16S* | *rdxA* | *gyrA* |
| HP-01 | 85 | 143 | N.D. | N.D. | N.D. | N.D. |
| HP+01 | 99 | 80 | 2,021 | 1,004 | 120 | 75 |
| HP+02 | 128 | 95 | 1,480 | 655 | 281 | 63 |
| HP+03 | 84 | 143 | 2,096 | 774 | 1,238 | 60 |
